# TAVR vs. SAVR in Patients With Severe Aortic Stenosis and Chronic Kidney Disease Undergoing Dialysis: A Comprehensive Meta‐Analysis

**DOI:** 10.1155/crp/9451268

**Published:** 2026-06-19

**Authors:** Maneeth Mylavarapu, Madiha Kiyani, Niharika Tanwar, Meghana Reddy, Israel Garcia, Nithin Karnan, Nidhi Laxminarayan Rao, Lakshmi Sai Meghana Kodali, Nimisha Borra

**Affiliations:** ^1^ Department of Cardiology, Endeavor Health Cardiovascular Institute, Endeavor Health Glenbrook Hospital, Glenview, Illinois, 60026, USA; ^2^ Division of Cardiology, Department of Medicine, University of Chicago Pritzker School of Medicine, Chicago, Illinois, 60637, USA, uchicago.edu; ^3^ Department of Internal Medicine, MedStar Health, Georgetown University, Washington, District of Columbia, 20007, USA, georgetown.edu; ^4^ Department of Internal Medicine, Advocate Illinois Masonic Medical Center, Chicago, Illinois, 60657, USA, advocatehealth.com; ^5^ Department of Internal Medicine, Mysore Medical College & Research Institute, Mysuru, Karnataka, 570001, India, mysoremedicalcollege.com; ^6^ Department of Family Medicine, University of Southern California School of Medicine, Los Angeles, California, 90033, USA, usc.edu; ^7^ Department of Internal Medicine, MercyOne North Iowa Medical Center, Mason City, Iowa, 50401, USA; ^8^ Department of Internal Medicine, Adventist Health Bakersfield, Bakersfield, California, 93301, USA, adventisthealth.org; ^9^ Department of Public Health & Health Sciences, University of Michigan - Flint, Flint, Michigan, 48502, USA; ^10^ Department of Internal Medicine, Kamineni Academy of Medical Sciences & Research Center, Hyderabad, Telangana, 500074, India

**Keywords:** chronic kidney disease, dialysis, end stage renal disease, meta-analysis, severe aortic stenosis, surgical aortic valve replacement, transcatheter aortic valve replacement

## Abstract

**Background:**

Patients with chronic kidney disease (CKD) undergoing dialysis who also suffer from severe aortic stenosis (AS) present a complex management challenge. Both transcatheter aortic valve replacement (TAVR) and surgical aortic valve replacement (SAVR) are treatment options, but comparative outcomes in this specific, high‐risk population continue to remain unclear.

**Aim:**

This study aims to compare TAVR and SAVR postoperative clinical outcomes in patients with severe AS and CKD undergoing dialysis.

**Methods:**

According to PRISMA guidelines, a comprehensive search was conducted across various databases such as PubMed, EMBASE, Scopus, and Google Scholar. Original studies that compared the clinical outcomes between TAVR and SAVR in patients with severe AS and CKD undergoing dialysis were included in the study.

**Results:**

Ten studies, all retrospective, involving 28,625 (14,625 TAVR and 14,000 SAVR) patients with severe AS and CKD undergoing dialysis who underwent TAVR or SAVR were included in this study. Patients who underwent TAVR had significantly lower odds of in‐hospital mortality (OR 0.49; 0.29, 0.84; *p* = 0.01) and shorter length of stay (LOS) (SMD −2.59; 95% CI −5.04, −0.14; *p* ≤ 0.04). However, the TAVR group had significantly higher odds of permanent pacemaker implantation (OR 2.25; 95% CI 1.71–2.94; *p* < 0.00001).

**Conclusion:**

In patients with severe AS and CKD undergoing dialysis, TAVR is associated with lower in‐hospital mortality and shorter LOS, suggesting a favorable early safety profile and recovery in this population.

## 1. Introduction

Aortic stenosis (AS) is the most prevalent valvular heart disease in developed countries, primarily affecting the elderly due to the progressive calcific degeneration of the aortic valve [1–3]. Severe AS is defined by an aortic valve area ≤ 1.0 cm^2^, a mean transvalvular gradient ≥ 40 mmHg, or a peak velocity ≥ 4.0 m/s [3]. The prevalence of AS increases with age, affecting approximately 2% of individuals over 65 years and up to 9.8% of those aged 80–89 years [2]. Severe symptomatic AS, if left untreated, carries a grim prognosis, with a 2‐year mortality rate of 50%–60% and a 3‐year survival rate of less than 30% [4].

The relationship between AS and chronic kidney disease (CKD) is complex, seemingly synergistic, and continues to remain a significant clinical challenge. Characterized by an estimated glomerular filtration rate (eGFR) < 60 mL/min/1.73 m^2^ or the presence of kidney damage for over three months, CKD is a prevalent comorbidity in the AS population [5]. While CKD affects approximately 35.5 million U.S. adults (14%) [6], the burden of AS is notably higher among those who progress to end‐stage kidney disease (ESKD). In this cohort, the prevalence of AS is estimated to be between 6% and 13% [7]. Beyond the shared epidemiological trends, the presence of CKD accelerates the progression of AS, with the aortic valve area decreasing at an estimated rate of 0.2 cm^2^ per year in CKD patients, compared to 0.1 cm^2^ per year in those without CKD [8]. This accelerated progression is driven by a distinct pathophysiological environment; dysregulated mineral metabolism, chronic systemic inflammation, and disordered calcium–phosphate homeostasis collectively facilitate premature and aggressive vascular and valvular calcification [9–12].

The coexistence of AS and CKD significantly exacerbates adverse clinical outcomes, creating a high‐risk phenotype characterized by increased cardiovascular events and all‐cause mortality [13]. Clinical management is frequently hindered by a heightened risk of perioperative complications and the characteristically rapid progression of valvular narrowing in this population. Data from the United States Renal Data System (USRDS) indicate that the burden remains substantial; approximately 7% of patients initiating dialysis present with moderate‐to‐severe AS, with prevalence increasing in direct correlation with dialysis vintage [14]. For these individuals, the presence of AS carries a nearly two‐fold increase in both all‐cause and cardiovascular mortality compared to those without valvular disease.

While surgical aortic valve replacement (SAVR) has traditionally served as the definitive treatment for severe AS, transcatheter aortic valve replacement (TAVR) has emerged as a transformative, less invasive alternative, particularly for high‐risk or inoperable cohorts. Recent comparative studies in the CKD population have yielded nuanced results. A meta‐analysis by Makki et al. observed in patients with CKD and high surgical risk, TAVR was associated with increased risk of short‐term and long‐term mortality [13]. Conversely, recent studies have reported that TAVR significantly mitigated the risks of in‐hospital mortality, stroke, and postoperative acute kidney injury (AKI) in patients with AS and CKD [15, 16].

Despite these preliminary insights, specific guidelines for AS in CKD are lacking, and CKD patients remain systematically underrepresented in major randomized controlled trials (RCTs), leaving a critical void in the evidence base required to guide clinical decision‐making [17]. Furthermore, the prognostic significance of CKD in severe AS is not fully understood, and evidence on AS progression in patients with CKD requiring it is contradictory [18]. Consequently, the optimal intervention strategy for this complex population remains a subject of ongoing debate. This comprehensive meta‐analysis aims to synthesize the existing evidence comparing the postoperative outcomes of TAVR and SAVR specifically in patients with comorbid severe AS and CKD requiring dialysis. By evaluating high‐impact endpoints, including in‐hospital mortality and length of hospitalization stay, this study seeks to provide the comparative clarity necessary to optimize individualized care and improve survival in this high‐risk population.

## 2. Methods

As per the Preferred Reporting Items for Systematic Reviews and Meta‐analyses (PRISMA) guidelines [19], a comprehensive literature search was conducted in several prominent reliable databases, including PubMed/MEDLINE, Cochrane, EMBASE, Science Direct, and Google Scholar. Subject headings and keywords for “transaortic catheter valve replacement,” “surgical aortic valve replacement,” “aortic valve/surgery,” “chronic kidney disease,” and “dialysis” were used along with appropriate Boolean operators. Using the snowballing method, the references of the selected studies were also examined to verify the comprehensiveness of the search. The search strategy utilized for the study is outlined in Supporting File S1. Studies that compared the clinical outcomes of adult patients with severe AS and CKD on dialysis who underwent TAVR or SAVR were included in our analysis. A detailed list of inclusion and exclusion criteria was outlined in Supporting File S2. Screening of the title and abstract was done independently by two reviewers, Israel Garcia and Niharika Tanwar, and conflicts were resolved by a third reviewer, Madiha Kiyani. Full‐text screening was done independently by two reviewers, Nithin Karnan and Meghana Reddy, and conflicts were resolved by a third reviewer, Maneeth Mylavarapu. The protocol of the study was registered in Open Science Framework (OSF) registry (Id: OSF.IO/4QD6R).

The risk of bias assessment was conducted using the adaptations of the Newcastle–Ottawa Scale (NOS) [20]. Outcomes are divided into in‐hospital outcomes, consisting of in‐hospital mortality and overall length of hospitalization, and other clinical outcomes, including postoperative stroke, major vascular complications, i.e., major vascular damage (MVD), and permanent pacemaker implantation (PPMI). Binary random effects were used to estimate the odds ratio (OR), and continuous random effects were used to estimate the mean differences (MD). Heterogeneity was assessed using the chi‐squared (*χ* ^2^) test and I^2^ statistics. The I^2^ statistic was interpreted as follows: < 25% (low heterogeneity), 25%–50% (moderate heterogeneity), and > 50% (high heterogeneity). Leave‐one‐out (LOO) sensitivity analyses were performed to evaluate the robustness of the pooled estimates. Funnel plots were utilized to visually evaluate publication bias; additionally, Egger’s regression test was performed to provide a quantitative assessment of plot asymmetry, with a *p* value < 0.05 indicating significant publication bias. All the statistical analyses were performed using the Review Manager (RevMan) version 5.4.1. A *p* value < 0.05 was considered to be statistically significant.

## 3. Results

**FIGURE 1:**
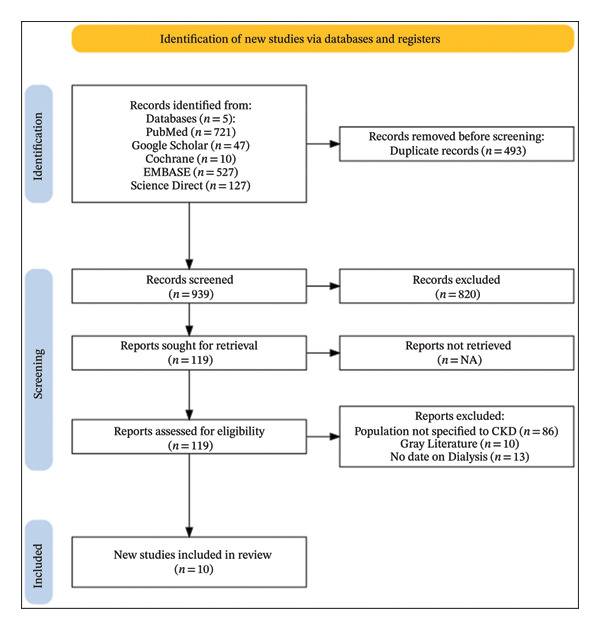
PRISMA flowchart of the study selection process.

In total, 10 studies (all retrospective) [21–30] with 28,625 patients with severe AS and CKD on dialysis who either underwent TAVR (14,625; 51.1%) or SAVR (14,000; 48.9%) are included in our analysis. Out of 10, 9 studies were conducted in the United States [22–30], and 1 in Europe [21]. The studies were subgrouped into ‘ESRD’ and ‘non‐ESRD’ groups based on the advanced stage of the CKD. Table 1 outlines the baseline characteristics of the included studies, and Figure 1 depicts the study selection process [31].

**TABLE 1:** Baseline characteristics of included studies.

Study	Nationality	Study type	CKD staging	TAVR/SAVR, *n*
Kobin et al.	United States	Retrospective propensity‐matched	CKD on Dialysis	194/194
Alquhtani et al.	United States	Retrospective propensity‐matched	CKD on Dialysis	197/197
Bhise et al.	United States	Retrospective propensity‐matched	ESRD on Dialysis	119/244
Condado et al.	United States	Retrospective observational	ESRD on Dialysis	30/30
Alkhalil et al.	United States	Retrospective propensity‐matched	CKD on Dialysis; ESRD on Dialysis	175/175
Ando et al.	United States	Retrospective observational	CKD on Dialysis	5,731/6,491
Färber et al.	Germany	Retrospective propensity‐matched	CKD on Dialysis	661/457
Khan et al.	United States	Retrospective propensity‐matched	ESRD on Dialysis	1,065/654
Mentias et al.	United States	Retrospective propensity‐matched	ESRD on Dialysis	4,130/2,565
Sanaiha et al.	United States	Retrospective observational	CKD (4–5) on Dialysis; ESRD on Dialysis	2,323/2,993

*Note:* CKD, chronic kidney disease; ESRD, end‐stage renal disease; n, number; SAVR, surgical aortic valve replacement; TAVR, transcatheter aortic valve replacement.

### 3.1. In‐Hospital Outcomes

**FIGURE 2:**
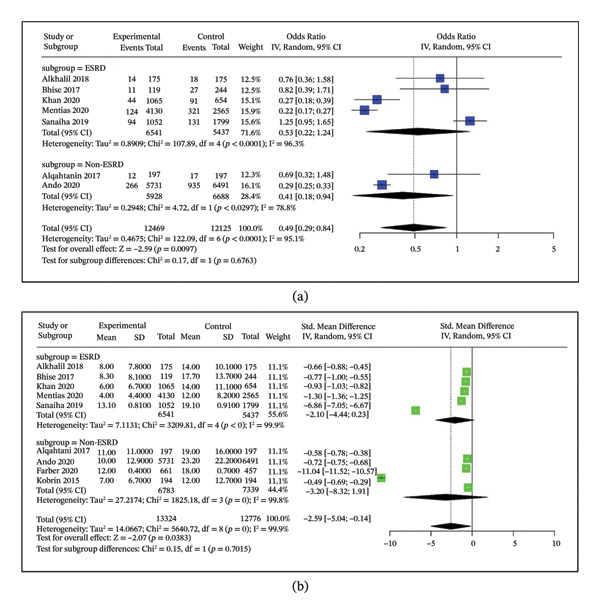
In‐hospital outcomes: (a) in‐hospital mortality and (b) length of stay (LOS).

TAVR was associated with a significant reduction in in‐hospital mortality compared to SAVR (OR 0.49; 95% CI 0.29–0.84; *p* = 0.01). However, in the ESRD subgroup, the mortality benefit for TAVR did not reach statistical significance (OR 0.53; 95% CI 0.22–1.24; *p* = 0.14) (Figure 2). Regarding length of stay (LOS), TAVR significantly reduced the LOS compared to SAVR (SMD −2.59; 95% CI −5.04, −0.14; *p* = 0.04). However, the reduction was greater in the non‐ESRD cohort compared to the ESRD cohort (3.2 vs 2.1 days) (Figure 2).

### 3.2. Other Clinical Outcomes

**FIGURE 3:**
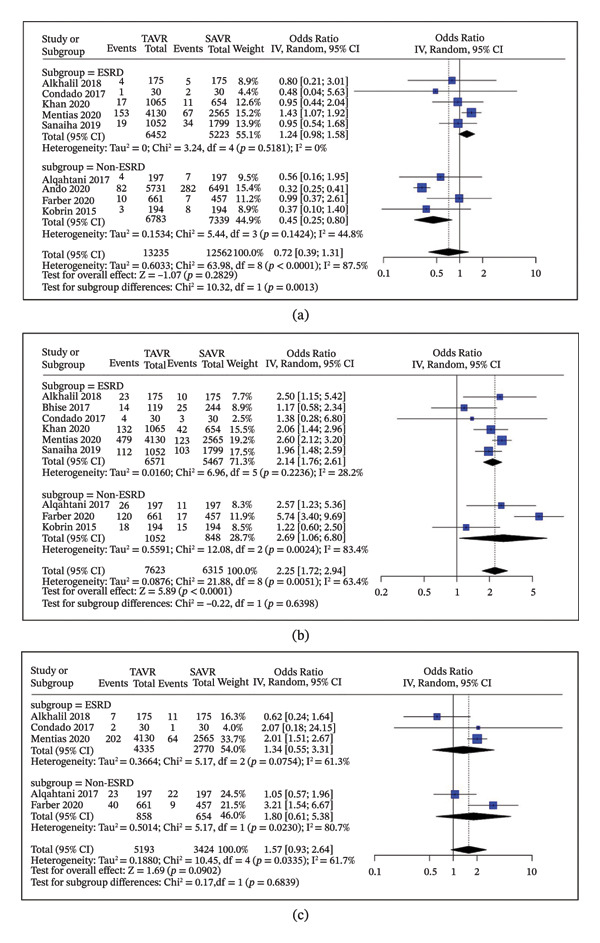
Other clinical outcomes: (a) postoperative stroke, (b) permanent pacemaker implantation (PPMI), and (c) major vascular damage (MVD).

TAVR significantly increased the odds of PPMI compared to SAVR (OR 2.25; 95% CI 1.71–2.94; *p* < 0.00001). This increase in odds was consistent in both the ESRD (OR 2.14) and non‐ESRD (OR 2.69) cohorts. No significant difference was observed between the two procedures regarding MVD (OR 1.57; 95% CI 0.93–2.64; *p* = 0.09) and postoperative stroke (OR 0.72; 95% CI 0.39–1.32; *p* = 0.28). Interestingly, however, in the non‐ESRD cohort, TAVR was associated with a significantly lower risk of stroke (OR 0.45; 95% CI 0.25–0.80; *p* = 0.006) (Figure 3).

### 3.3. Assessment of Heterogeneity and Bias

Our analyses revealed significant moderate to high heterogeneity in the majority of outcomes assessed, including in‐hospital mortality (I^2^ = 95%), LOS (I^2^ = 99%), and PPMI (I^2^ = 64%). Supporting File S3 outlines the results of the heterogeneity assessment. Regarding LOO sensitivity analyses, for in‐hospital mortality, the I^2^ remained consistently high across all subsets (ranging from 85.2% to 97.1%). For LOS, the I^2^ remained nearly stagnant at approximately 99%, regardless of which study was omitted. In PPMI, the removal of Farber 2020 [21] resulted in the I^2^ for the total population decreasing from 63.4% to 28.4%. For MVD, the I^2^ values fluctuated between 46.4% and 71.2% during the exclusion process. Supporting File S4 outlines the results of LOO sensitivity analyses.

On visual assessment of the funnel plots, in‐hospital mortality, postoperative stroke, PPMI, and MVD revealed a relatively symmetrical distribution of studies within the pseudo‐confidence intervals, suggesting a low likelihood of significant publication bias for these clinical endpoints. However, the funnel plot for LOS exhibited notable asymmetry, with several studies falling outside the expected distribution, likely reflecting the extreme heterogeneity (I^2^ = 99%) and varying institutional discharge protocols discussed previously. Supporting File S5 reports funnel plots for publication bias assessment.

Regarding risk of bias assessment, almost all of the included studies (7/10) [21–27] demonstrated a low risk across most domains for the majority of the literature. Supporting File S6 reports the risk of bias assessment of the included studies.

## 4. Discussion

The current meta‐analysis, encompassing 28,625 patients across 10 studies, provides a comprehensive evaluation of TAVR versus SAVR in the high‐risk population of patients with severe AS and comorbid CKD on dialysis. Our findings demonstrate that TAVR is associated with a significant reduction in the odds of in‐hospital mortality and a substantial reduction in the length of hospital stay compared to SAVR. These findings support existing guidelines suggesting TAVR as the preferred AVR procedure in older and high‐risk patients [32, 33].

The mortality benefit associated with TAVR appears to be modulated by the severity of renal impairment, as the statistical significance was attenuated in the ESRD subgroup, aligning with the previous analyses [34]. The observed mortality benefit with TAVR aligns with the broader trend toward less invasive interventions for patients with significant multimorbidity. In patients with CKD, the surgical “hit” of SAVR, often involves cardiopulmonary bypass and systemic inflammatory response which can be particularly deleterious [35]. However, these findings are hypothesis‐generating and underscore the necessity for larger, dedicated studies to confirm if a true mortality benefit exists for this specific cohort.

Our results suggest that by avoiding sternotomy and bypass, TAVR mitigates these early perioperative risks. Interestingly, the loss of a statistically significant mortality benefit in the ESRD‐specific subgroup suggests that as renal disease reaches its terminal stage, the physiological frailty and dysregulated metabolic state may diminish the relative advantages of a less invasive approach, or perhaps reflect the inherent competitive risk of mortality in dialysis patients [34].

The significant reduction in LOS (≈2.5 days) across all cohorts is of profound clinical and economic importance. Dialysis patients are particularly susceptible to hospital‐acquired infections and volume imbalances; therefore, a shorter convalescence period likely contributes to the lower in‐hospital mortality observed in the TAVR group. The fact that the LOS reduction was slightly more pronounced in the non‐ESRD cohort may reflect the increased complexity and slower recovery times inherent to patients already dependent on maintenance dialysis. However, these findings must be interpreted with caution. LOO sensitivity analyses confirmed that this heterogeneity was remarkably stable for these primary endpoints, i.e., mortality and LOS, suggesting that the high variance is a systemic feature of the existing literature, likely stemming from heterogeneous institutional practices, rather than the influence of a single outlier study. Specifically, in regard to LOS, in the absence of standardized, prospective discharge criteria across the included studies, the variability in LOS may be partially confounded by heterogeneous institutional practices and healthcare delivery models rather than purely representing accelerated clinical recovery.

Our analysis revealed a nuanced stroke profile. While the overall stroke risk did not differ significantly between procedures, TAVR offered a clear protective effect in the non‐ESRD cohort (OR 0.45). This may be attributed to improvement in valve technologies, patient selection criteria, and operator experience [36]. However, the consistent increase in PPMI following TAVR (OR 2.25) remains a notable drawback. This is likely due to the mechanical interaction between the transcatheter valve frame and the conduction system, a risk that is potentially exacerbated in CKD patients due to the high prevalence of pre‐existing conduction tissue calcification [37].

Although subgroup analyses (ESRD vs. non‐ESRD) provide preliminary insights, these findings may be underpowered to detect true differences in mortality and should be viewed as hypothesis‐generating. Additionally, while TAVR was associated with a shorter LOS, the clinical significance of this finding must be interpreted with caution, as standardized discharge criteria were not uniform across the included retrospective studies. Furthermore, given the I^2^ values exceeding 90% for primary endpoints, these pooled estimates should be interpreted as suggestive of a trend rather than a definitive effect size.

### 4.1. Integration With the Landmark Clinical Trial Landscape

Direct comparison between our meta‐analysis and the landmark RCTs is inherently limited by variations in study design, patient characteristics, and evolving valve technologies. Nonetheless, when framed within the context of existing TAVR literature and prior clinical trials [38–40], the mortality benefits observed in our dialysis‐dependent cohort are higher than those reported in the extreme surgical risk TAVR trials. Our findings underscore a critical gap in the existing literature: although ESRD patients were systematically excluded from pivotal RCTs, they represent an “extreme‐risk” cohort whose outcomes may exceed the mortality thresholds of the previously studied inoperable populations.

### 4.2. Limitations

Several limitations impact the findings of this meta‐analysis. Primarily, the reliance on retrospective data introduces inherent selection bias. While most included studies utilized propensity‐score matching to align baseline characteristics, this methodology cannot account for unmeasured or residual confounding. Crucially, baseline risk metrics, such as Society of Thoracic Surgeons (STS) scores, objective frailty indices, and dialysis vintage, were not quantitatively synthesized due to inconsistent reporting across the primary literature. These factors significantly influence procedural selection, and their absence suggests that the observed outcomes should be interpreted as associations rather than definitive causal effects.

The substantial heterogeneity observed (I^2^> 90% for primary endpoints) further restricts the generalizability of these results. In particular, the observed reduction in LOS likely reflects variations in institutional discharge protocols and healthcare delivery models rather than purely clinical recovery rates, as standardized discharge criteria were not uniform across the studies. Additionally, while Egger’s regression test was used to assess publication bias, the reliance on published retrospective data remains a constraint. Finally, the lack of long‐term follow‐up data in several studies limits our ability to evaluate the durability of the TAVR benefit beyond the acute hospital phase. Given these constraints, our ESRD subgroup analysis is hypothesis‐generating and underscores the urgent need for RCTs to establish definitive clinical guidelines for this extreme‐risk population.

## 5. Conclusion

In patients with severe AS on dialysis, TAVR demonstrates a superior short‐term safety profile with lower in‐hospital mortality and shorter hospitalizations compared to SAVR. However, these benefits are tempered by a significant increase in PPMI and a lack of clear mortality benefit in the ESRD‐specific subgroup. Given the observational nature of current data and high heterogeneity, these findings highlight the urgent need for RCTs to establish long‐term guidelines for this extreme‐risk population.

## Author Contributions

Maneeth Mylavarapu, Niharika Tanwar, and Israel Garcia has made significant contributions in terms of conceptualization; Maneeth Mylavarapu and Madiha Kiyani has made significant contributions in terms of editorial work; Lakshmi Sai Meghana Kodali and Maneeth Mylavarapu performed data curation, visualization, and interpretation. Maneeth Mylavarapu and Madiha Kiyani contributed equally to this article, and hence, they are the co‐first authors of this manuscript.

## Funding

No funding was received for this manuscript.

## Disclosure

All the authors wrote, reviewed and edited the manuscript; and all authors have read and approved the final manuscript.

## Conflicts of Interest

The authors declare no conflicts of interest.

## Supporting Information

Additional supporting information can be found online in the Supporting Information section.

## Supporting information


**Supporting Information** Supporting File S1: Search Strategy. Outlines the explicit, systematic search queries utilized across major biomedical databases (including PubMed/MEDLINE, EMBASE, the Cochrane Library, Science Direct, and Google Scholar) to identify clinical studies comparing transcatheter aortic valve replacement (TAVR) vs. surgical aortic valve replacement (SAVR) in patients requiring dialysis. Supporting File S2: Inclusion and Exclusion Criteria. Details the precise PICOS framework used for study selection, defining target patient populations (adults with severe aortic stenosis and chronic kidney disease on dialysis), valid interventions, and evaluated clinical endpoints (such as in‐hospital mortality, length of stay, postoperative stroke, permanent pacemaker implantation, and major vascular damage). Supporting File S3: Assessment of Heterogeneity. Tabulates the statistical heterogeneity evaluations (I^2^, chi2, tau2, and *p* values) across all clinical outcomes, stratified into end‐stage renal disease (ESRD), non‐ESRD, and overall patient cohorts. Supporting File S4: Leave‐One‐Out Sensitivity Analysis. Presents the iterative reliability analysis performed by systematically excluding individual datasets to observe their isolated impacts on overall cohort heterogeneity (I^2^) across all major clinical endpoints. Supporting File S5: Publication Assessment. Provides visual funnel plot representations (S5a through S5e) tracking standard error against the calculated treatment effect metrics (odds ratio and standardized mean difference) to assess potential publication bias across the included literature. Supporting File S6: Risk of Bias Assessment. Evaluates internal study quality utilizing the Newcastle–Ottawa Scale (NOS) and key bias domains (such as allocation concealment, selection bias, and blinding), demonstrating that the overall study portfolio exhibits high methodological quality with scores ranging between 6 and 8.

## Data Availability

The data that support the findings of this study are available from the corresponding author upon reasonable request.
