# Supplementary material for: TAVR vs. SAVR in Patients With Severe Aortic Stenosis and Chronic Kidney Disease Undergoing Dialysis: A Comprehensive Meta‐Analysis
Source: Cardiol Res Pract. 2026 Jun 19;2026:9451268. doi: 10.1155/crp/9451268 (PMC13280660; doi:10.1155/crp/9451268)
Supplement: Supplementary file 1 — Supporting Information Supporting File S1: Search Strategy. Outlines the explicit, systematic search queries utilized across major biomedical databases (including PubMed/MEDLINE, EMBASE, the Cochrane Library, Science Direct, and Google Scholar) to identify clinical studies comparing transcatheter aortic valve replacement (TAVR) vs. surgical aortic valve replacement (SAVR) in patients requiring dialysis. Supporting File S2: Inclusion and Exclusion Criteria. Details the precise PICOS framework used for study selection, defining target patient populations (adults with severe aortic stenosis and chronic kidney disease on dialysis), valid interventions, and evaluated clinical endpoints (such as in‐hospital mortality, length of stay, postoperative stroke, permanent pacemaker implantation, and major vascular damage). Supporting File S3: Assessment of Heterogeneity. Tabulates the statistical heterogeneity evaluations (I2, chi2, tau2, and p values) across all clinical outcomes, stratified into end‐stage renal disease (ESRD), non‐ESRD, and overall patient cohorts. Supporting File S4: Leave‐One‐Out Sensitivity Analysis. Presents the iterative reliability analysis performed by systematically excluding individual datasets to observe their isolated impacts on overall cohort heterogeneity (I2) across all major clinical endpoints. Supporting File S5: Publication Assessment. Provides visual funnel plot representations (S5a through S5e) tracking standard error against the calculated treatment effect metrics (odds ratio and standardized mean difference) to assess potential publication bias across the included literature. Supporting File S6: Risk of Bias Assessment. Evaluates internal study quality utilizing the Newcastle–Ottawa Scale (NOS) and key bias domains (such as allocation concealment, selection bias, and blinding), demonstrating that the overall study portfolio exhibits high methodological quality with scores ranging between 6 and 8. [file CRP-2026-9451268-s001.docx]

**Supplementary File S1. Search Strategy**

| **Database** | **Search Strategy** |
| --- | --- |
| PubMed/MEDLINE | (("Aortic Valve Replacement"[Mesh] OR "Transcatheter Aortic Valve Replacement"[Mesh] OR "TAVR" OR "TAVI" OR "Surgical Aortic Valve Replacement" OR "SAVR") AND ("Chronic Kidney Disease"[Mesh] OR "Renal Insufficiency, Chronic"[Mesh] OR "Kidney Failure, Chronic"[Mesh] OR "Renal Dialysis"[Mesh] OR "chronic kidney disease" OR "renal impairment" OR "end stage renal disease" OR "ESRD" OR "dialysis")) |
| EMBASE | ('aortic valve replacement'/exp OR 'transcatheter aortic valve replacement'/exp OR 'TAVR' OR 'TAVI' OR 'surgical aortic valve replacement' OR 'SAVR') AND ('chronic renal insufficiency'/exp OR 'dialysis'/exp OR 'chronic kidney disease' OR 'renal impairment' OR 'end stage renal disease' OR 'ESRD' OR 'hemodialysis' OR 'peritoneal dialysis') |
| Cochrane Library | ([MeSH descriptor: Aortic Valve Replacement] OR "TAVR" OR "TAVI" OR "SAVR") AND ([MeSH descriptor: Chronic Kidney Disease] OR [MeSH descriptor: Renal Dialysis] OR "chronic kidney disease" OR "renal impairment" OR "end stage renal disease" OR "dialysis") |
| ScienceDirect | ("Aortic Valve Replacement" OR "TAVR" OR "TAVI" OR "SAVR") AND ("Chronic Kidney Disease" OR "End Stage Renal Disease" OR "ESRD" OR "Dialysis") |
| Google Scholar | allintitle: "Transcatheter" OR "Surgical" "Aortic" "Stenosis" "Dialysis" OR "ESRD" |

**Supplementary File S2. Inclusion and Exclusion Criteria**

| **Category** | **Inclusion Criteria** | **Exclusion Criteria** |
| --- | --- | --- |
| **Study Design** | Studies comparing TAVR and SAVR in adult patients:   - Observational studies (cohort, case-control) - RCTs | - Case reports, case series, editorials, letters, reviews - Studies not comparing TAVR and SAVR |
| **Patient Population** | - Adults (≥18 years) with severe AS - Patients with pre-existing CKD (any stage) on dialysis | - Patients without severe AS or without CKD or with CKD but not on dialysis - Pediatric patients (<18 years) - Patients with AKI only |
| **Interventions** | - TAVR (any approach) - SAVR (any surgical technique) | - Studies not involving TAVR or SAVR - Studies involving only redo procedures |
| **Outcomes** | - Studies reporting Any in-hospital or clinical outcomes, including but not limited to, in-hospital mortality, LOS, stroke, MVD, and PPMI | - Studies not reporting any of the specified outcomes- Studies with incomplete or unavailable data |
| **Other** | - | - Studies with overlapping data or duplicate publications - Non-English studies |

**Legend**: AKI, Acute Kidney Injury; AS, Aortic Stenosis; CKD, Chronic Kidney Disease; ESRD, End-Stage Renal Disease; LOS, Length of Hospitalization Stay; MVD, Major Vascular Damage; PPMI, Permanent Pacemaker Insertion; RCT, Randomized Controlled Trial; SAVR, Surgical Aortic Valve Replacement; TAVR, Transcatheter Aortic Valve Replacement.

**Supplementary File S3. Assessment of Heterogeneity**

| **Outcome** | **Subgroup** | **I2 (%)** | **χ2** | **P-value (Heterogeneity)** | **τ2** |
| --- | --- | --- | --- | --- | --- |
| **In-hospital Mortality** | ESRD | 96.3% | 107.92 | < 0.00001 | 0.89 |
|  | Non-ESRD | 77.8% | 4.73 | 0.03 | 0.29 |
|  | Overall | 95.1% | 122.14 | < 0.00001 | 0.47 |
| **Length of Stay (LOS)** | ESRD | 99.9% | 149.54 | < 0.00001 | 1.96 |
|  | Non-ESRD | 99.8% | 490.8 | < 0.00001 | 22.47 |
|  | Overall | 99.8% | 640.38 | < 0.00001 | 0.7 |
| **Post-operative Stroke** | ESRD | 0% | 3.24 | 0.52 | 0 |
|  | Non-ESRD | 44.8% | 5.44 | 0.14 | 0.15 |
|  | Overall | 87.% | 64.2 | < 0.00001 | 0.61 |
| **PPMI** | ESRD | 28.2% | 6.99 | 0.22 | 0.02 |
|  | Non-ESRD | 83.4% | 12.3 | 0.002 | 0.57 |
|  | Overall | 63.44% | 22.08 | 0.005 | 0.09 |
| **Major Vascular Damage** | ESRD | 61.3% | 5.17 | 0.08 | 0.37 |
|  | Non-ESRD | 80.7% | 5.24 | 0.02 | 0.51 |
|  | Overall | 61.7% | 10.47 | 0.03 | 0.19 |

**Legend:** ESRD, End-Stage Renal Disease; LOS, Length of Stay; PPMI, Permanent Pacemaker Implantation

**Supplementary File S4. Leave One Out Sensitivity Analysis**

| **Outcome** | **Study Removed** | **I2% (ESRD)** | **I2% (Non-ESRD)** | **I2% (Total)** |
| --- | --- | --- | --- | --- |
| **In hospital Mortality** | All studies included | 96.3 | 77.8 | 95.1 |
|  | Alkhalil 2018 | 97.1 | - | 95.7 |
|  | Bhise 2017 | 97 | - | 95.7 |
|  | Khan 2020 | 97.1 | - | 95.8 |
|  | Mentias 2020 | 92.9 | - | 94.9 |
|  | Sanaiha 2019 | 85.2 |  | 81 |
|  | Alqahtani 2017 | - | N/A | 95.8 |
|  | Ando 2020 | - | N/A | 95.4 |
| **Length of Stay (LOS)** | All studies included | 99.9 | 99.8 | 99.9 |
|  | Alkhalil 2018 | 99.8 | - | 99.8 |
|  | Bhise 2017 | 99.2 | - | 98 |
|  | Khan 2020 | 99.8 | - | 99.8 |
|  | Mentias 2020 | 99.6 | - | 99.6 |
|  | Sanaiha 2019 | 96.2 | - | 99.7 |
|  | Alqahtani 2017 | - | 99.2 | 99.4 |
|  | Ando 2020 | - | 99.5 | 99.4 |
|  | Farber 2020 | - | 67.1 | 99.8 |
|  | Kobrin 2015 | - | 95.6 | 98.2 |
| **Post-operative Stroke** | All studies included | 0 | 44.8 | 87.5 |
|  | Alkhalil 2018 | - | - | 87.5 |
|  | Condado 2017 | - | - | 87.5 |
|  | Khan 2020 | - | - | 87.5 |
|  | Mentias 2020 | - | - | 87.5 |
|  | Sanaiha 2019 | - | - | 87.5 |
|  | Alqahtani 2017 | - | 44.2 | 86 |
|  | Ando 2020 | - | 0 | 10.3 |
|  | Farber 2020 | - | 41 | 87.1 |
|  | Kobrin 2015 | - | 63.2 | 88.9 |
| **PPMI** | All studies included | 28.2 | 83.4 | 63.4 |
|  | Alkhalil 2018 | 41.9 | - | 68 |
|  | Bhise 2017 | 0 | - | 68.9 |
|  | Condado 2017 | 39.5 | - | 67.4 |
|  | Khan 2020 | 40.6 | - | 67.2 |
|  | Mentias 2020 | 0 | - | 65.1 |
|  | Sanaiha 2019 | 31.2 | - | 64.8 |
|  | Alqahtani 2017 | - | 91.5 | 67.9 |
|  | Farber 2020 | - | 50.6 | 28.4 |
|  | Kobrin 2015 | - | 67.2 | 62.4 |
| **Major Vascular Damage** | All studies included | 61.3 | 80.7 | 61.7 |
|  | Alkhalil 2018 | 0 | - | 46.4 |
|  | Condado 2017 | 80.6 | - | 71.2 |
|  | Mentias 2020 | 0 | - | 64.7 |
|  | Alqahtani 2017 | - | N/A | 51.9 |
|  | Farber 2020 | - | N/A | 61.3 |

**Legend:** ESRD, End-Stage Renal Disease; LOS, Length of Stay; PPMI, Permanent Pacemaker Implantation; N/A, Not Applicable

**Supplementary File S5. Publication Assessment**

**S5a. In-hospital Mortality**

**
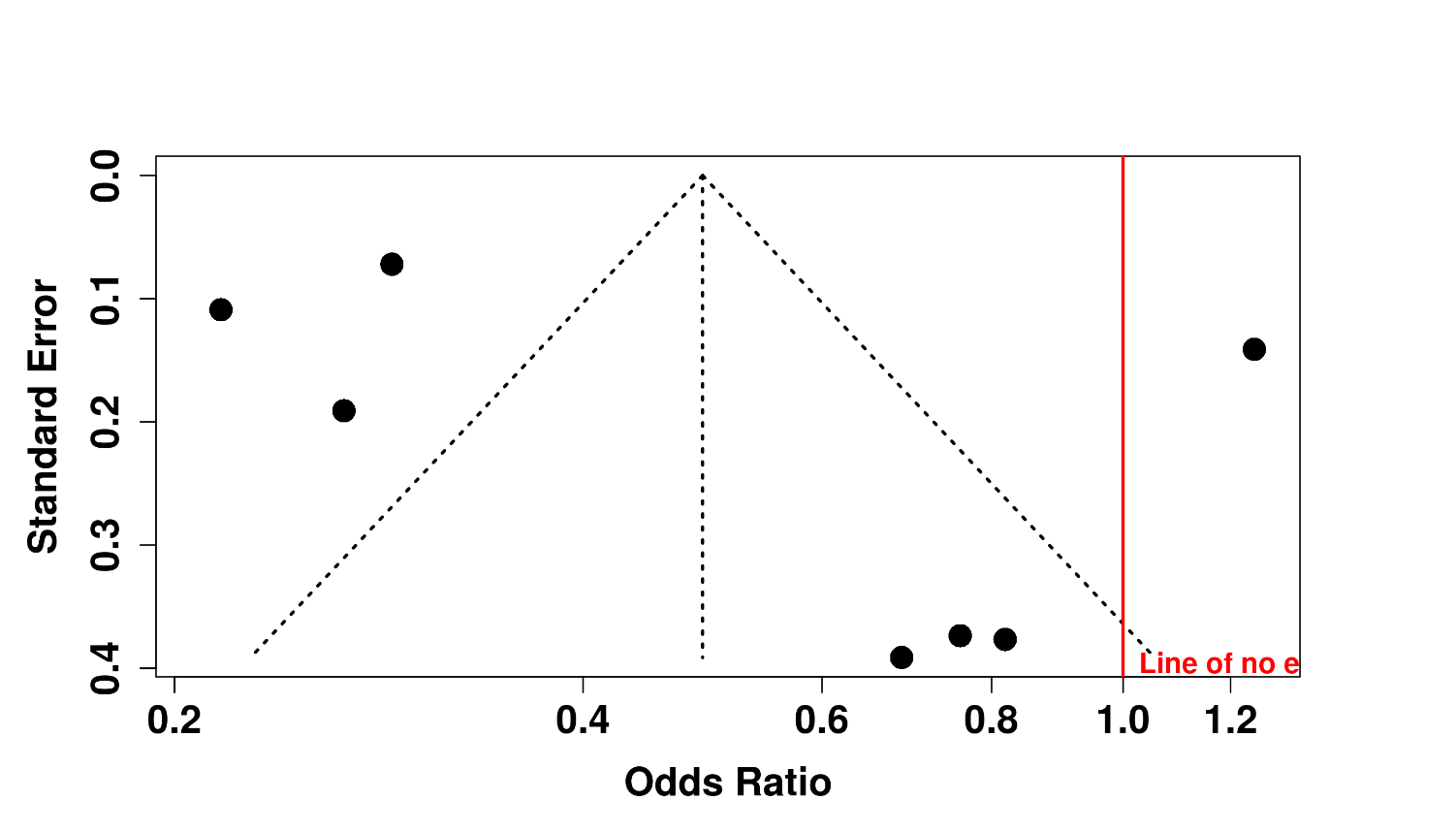
**

**S5b. Length of Stay (LOS)**

**
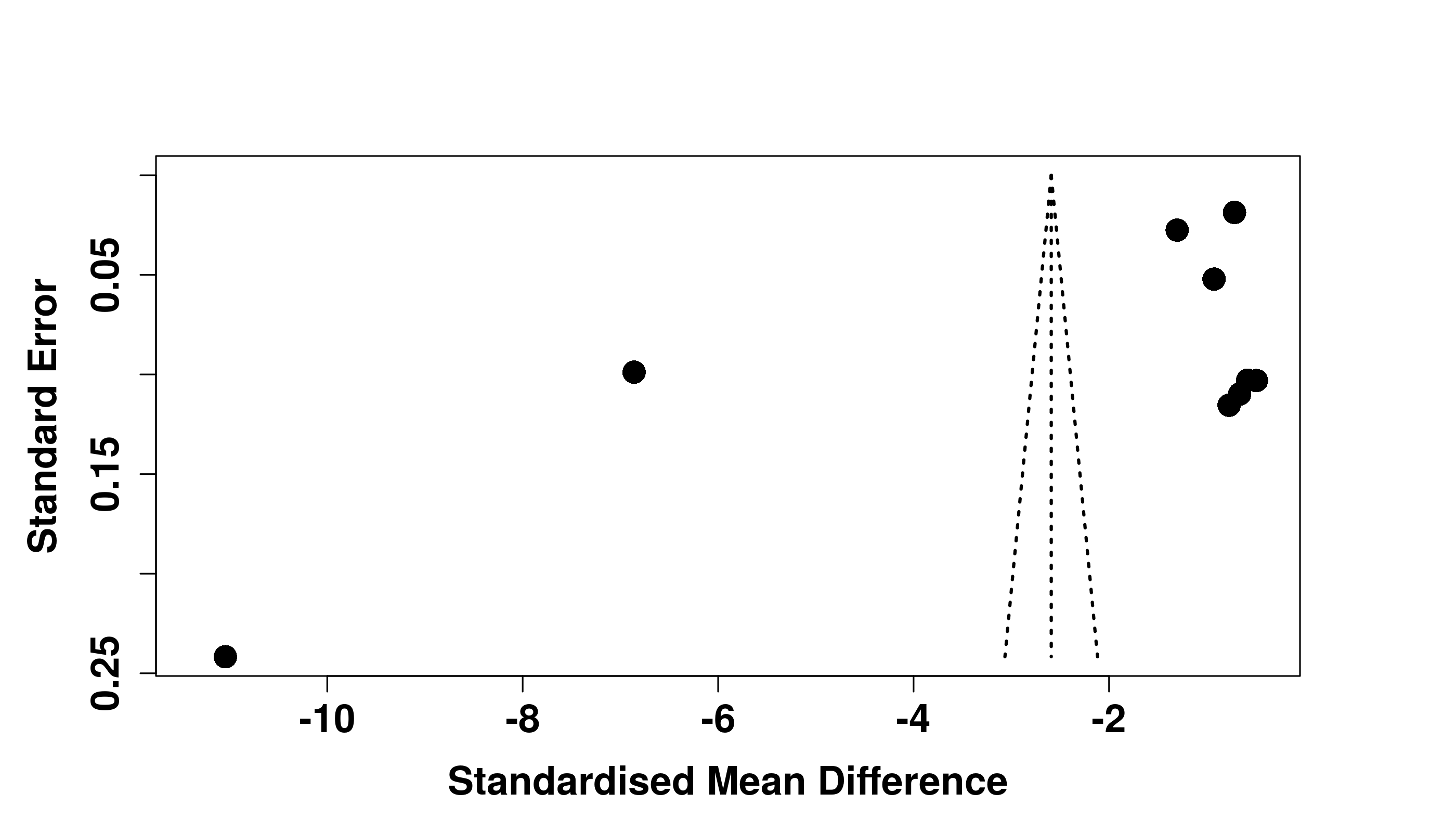
**

**S5c. Postoperative Stroke**

**
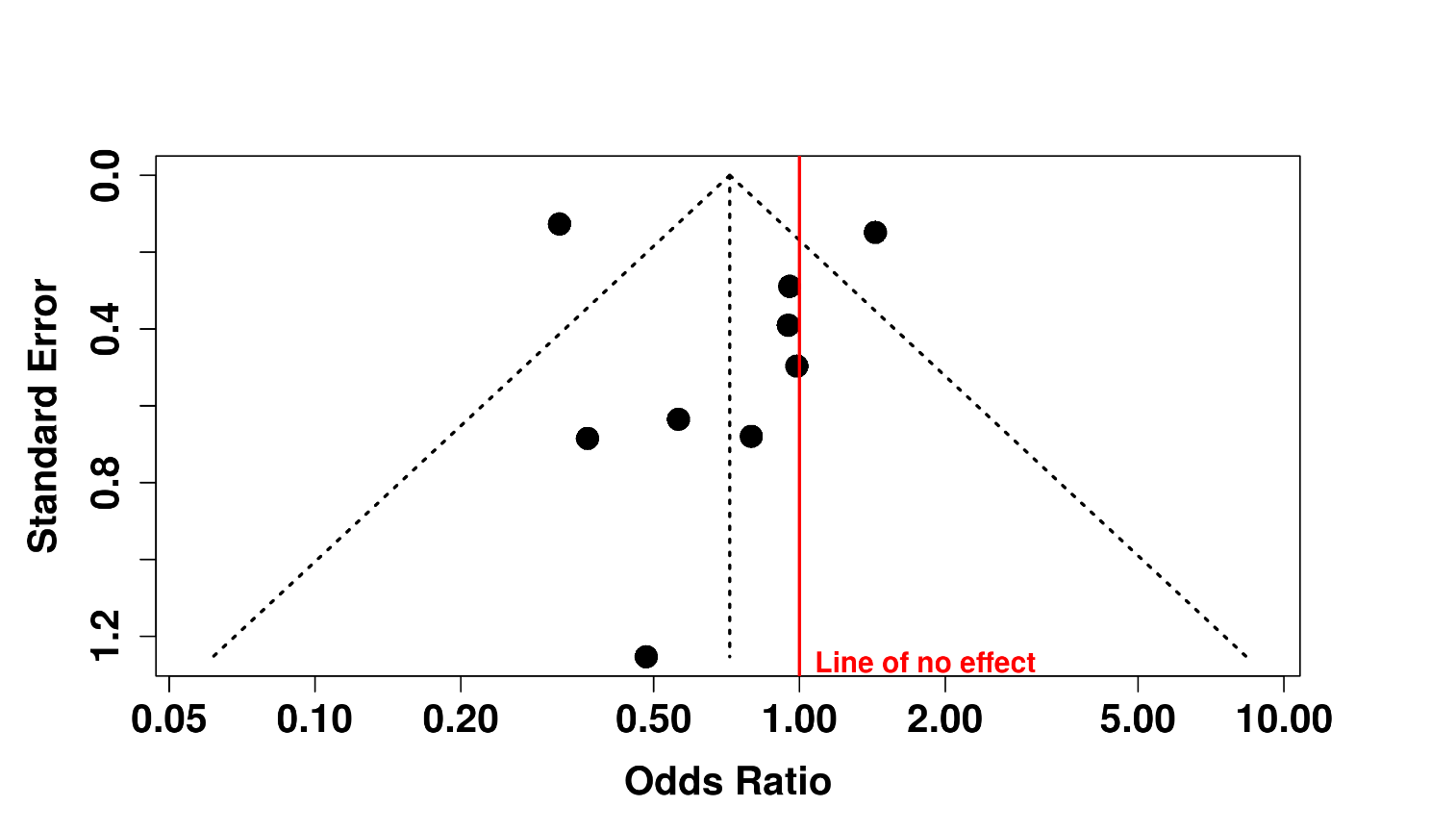
**

**S5d. Permanent Pacemaker Implantation (PPMI)**

**
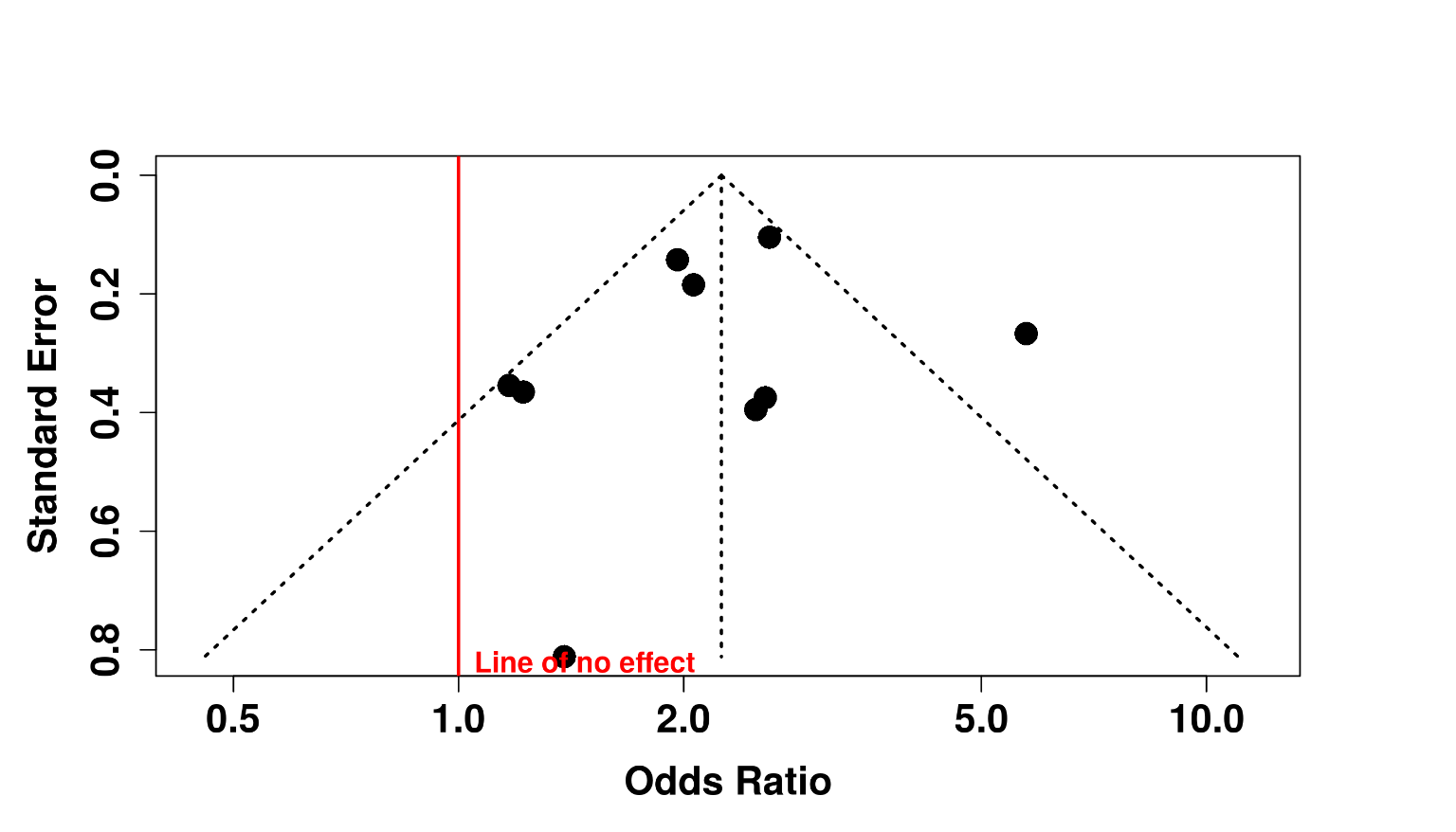
**

**S5e. Major Vascular Damage (MVD)**

**
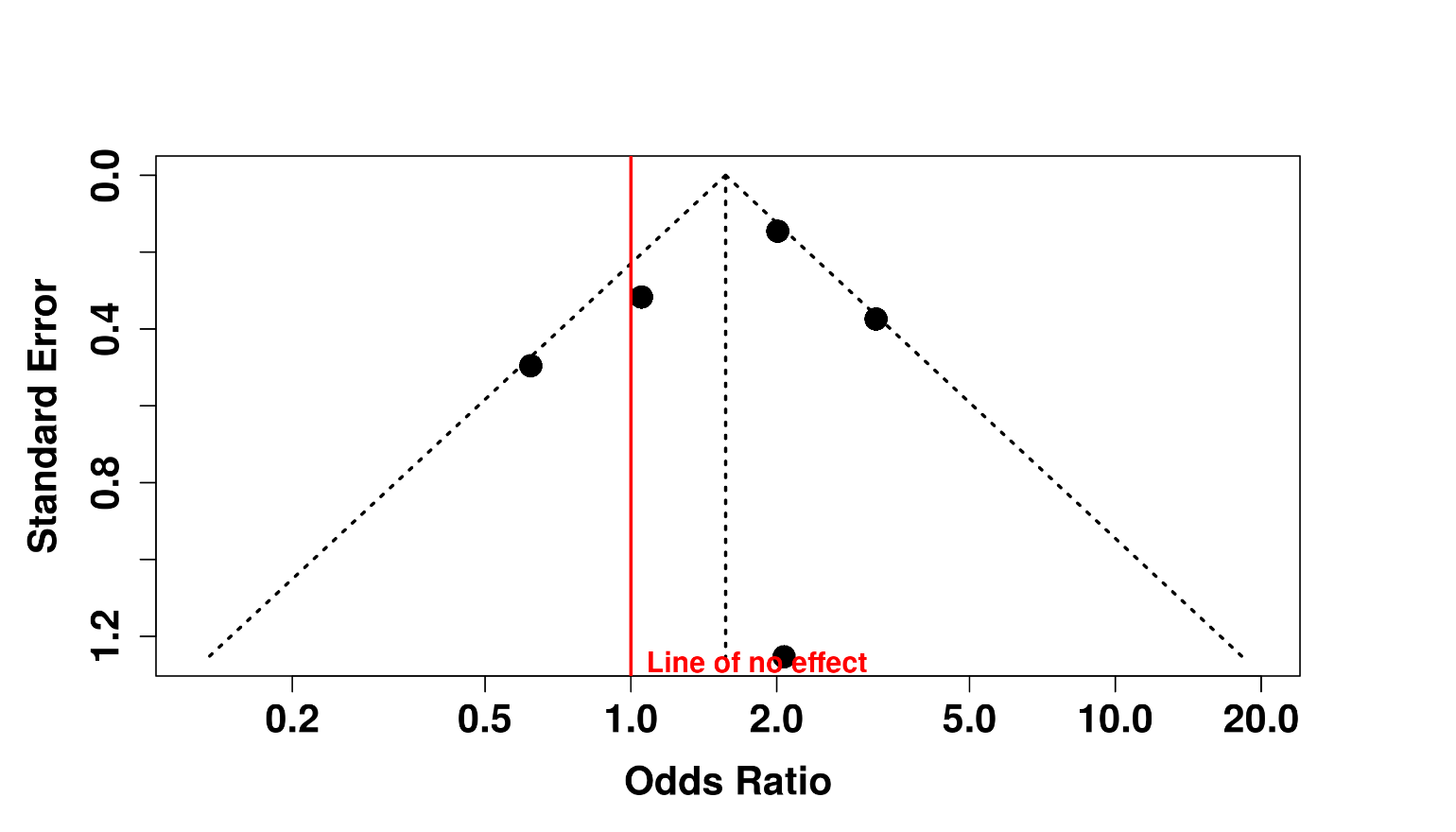
**

**Supplementary File S6. Risk of Bias Assessment**

| **Study** | **RS** | **AC** | **BPP** | **BOA** | **IOD** | **SR** | **OSB** | **OSQ** | **Study Quality** |
| --- | --- | --- | --- | --- | --- | --- | --- | --- | --- |
| **Kobin et al.** | Low | Low | Low | Low | Low | Low | Low | Low | NOS: 8 |
| **Alquhtani et al.** | Low | Low | Low | Low | Low | Low | Low | Low | NOS: 8 |
| **Bhise et al.** | Low | Low | Low | Low | Unclear | Low | Unclear | Unclear | NOS: 6 |
| **Condado et al.** | Low | Unclear | Unclear | Low | Low | Low | Low | Unclear | NOS: 6 |
| **Alkhalil et al.** | Low | Low | Low | Low | Low | Low | Low | Low | NOS: 8 |
| **Ando et al.** | Low | Unclear | Low | Unclear | Unclear | Low | Low | Unclear | NOS: 6 |
| **Färber et al.** | Low | Low | Low | Low | Low | Low | Low | Low | NOS: 8 |
| **Khan et al.** | Low | Low | Low | Low | Low | Low | Low | Unclear | NOS: 8 |
| **Mentias et al.** | Low | Low | Low | Low | Low | Low | Low | Unclear | NOS: 7 |
| **Sanaiha et al.** | Low | Low | Low | Low | Low | Low | Low | Unclear | NOS: 7 |

**Legend:** AC, allocation concealment; BOA, blinding of outcome assessment; BPP, blinding of participants and personnel; IOD, incomplete outcome data; NOS, Newcastle-Ottawa Scale; OSB, other sources of bias; OSQ, overall study quality; RSG, random sequence generation; SR, selective reporting
